# P-Type ATPase TAT-2 Negatively Regulates Monomethyl Branched-Chain Fatty Acid Mediated Function in Post-Embryonic Growth and Development in *C. elegans*

**DOI:** 10.1371/journal.pgen.1000589

**Published:** 2009-08-07

**Authors:** Emylie Seamen, Jennifer M. Blanchette, Min Han

**Affiliations:** 1 Howard Hughes Medical Institute, University of Colorado at Boulder, Boulder, Colorado, United States of America; 2 Department of Molecular, Cellular, and Developmental Biology, University of Colorado at Boulder, Boulder, Colorado, United States of America; University of California San Francisco, United States of America

## Abstract

Monomethyl branched-chain fatty acids (mmBCFAs) are essential for *Caenorhabditis elegans* growth and development. To identify factors acting downstream of mmBCFAs for their function in growth regulation, we conducted a genetic screen for suppressors of the L1 arrest that occurs in animals depleted of the 17-carbon mmBCFA C17ISO. Three of the suppressor mutations defined an unexpected player, the P-type ATPase TAT-2, which belongs to the flippase family of proteins that are implicated in mediating phospholipid bilayer asymmetry. We provide evidence that TAT-2, but not other TAT genes, has a specific role in antagonizing the regulatory activity of mmBCFAs in intestinal cells. Interestingly, we found that mutations in *tat-2* also suppress the lethality caused by inhibition of the first step in sphingolipid biosynthesis. We further showed that the fatty acid side-chains of glycosylceramides contain 20%–30% mmBCFAs and that this fraction is greatly diminished in the absence of mmBCFA biosynthesis. These results suggest a model in which a C17ISO-containing sphingolipid may mediate the regulatory functions of mmBCFAs and is negatively regulated by TAT-2 in intestinal cells. This work indicates a novel connection between a P-type ATPase and the critical regulatory function of a specific fatty acid.

## Author Summary

Fatty acids serve diverse functions in organisms, including roles at the cell membrane to coordinate cell signaling processes. Monomethyl branched-chain fatty acids (mmBCFAs) are a special type of fatty acid that is commonly present in animals. Because mmBCFAs are a small component of the total fatty acid pool, their functions have not been a major research focus and are largely unclear. We tackled the problem using the nematode *C. elegans*. Our laboratory previously found that without mmBCFAs, worms cannot develop normally and die. To understand how these obscure fatty acids perform such important roles, we searched for other factors involved in the process by conducting a mutagenesis screen to uncover mutant worms that can recover the ability to grow without the presence of mmBCFAs. We found several such mutations in a single gene that codes for a protein called TAT-2. TAT-2 is one of several poorly understood P-type ATPases that likely help maintain the proper lipid structure in cell membranes. Our work indicates that TAT-2 antagonizes the growth regulatory function of mmBCFAs in intestinal cells. Studies on how mmBCFAs and this protein functionally interact explore a novel, interesting, and important problem that is only beginning to be understood.

## Introduction

Lipids play many critical roles in cellular function ranging from providing structural support within cell membranes to mediating signaling events. The importance of the particular fatty acid constituents of complex lipids is only beginning to be understood. While extensive analyses have been conducted to elucidate the specific roles of straight-chain saturated and unsaturated fatty acids, there is little known about the roles of monomethyl branched-chain fatty acids (mmBCFAs) in animals. We have previously identified an essential role for mmBCFAs in regulating post-embryonic development in the model organism *C. elegans*, where the deletion of *elo-5*, a gene encoding a very long chain fatty acid (VLCFA) elongase, causes larval arrest and death. When the fatty acid composition of worms lacking functional ELO-5 was compared to that of wild-type worms, two detectable mmBCFAs (C15ISO and C17ISO) were missing. In addition, when the ELO-5 deficient strain was supplemented with exogenous C15ISO and C17ISO, the nematodes recovered from larval arrest and were able to proliferate similar to wild-type worms [1]. The L1 arrest that occurs in the absence of C17ISO is strikingly similar to the L1 diapause that occurs in the absence of food [2]. In both cases, the worms arrest at an early L1 stage, just after hatching and prior to the first M cell division, and the arrest is reversible upon restoration of the depleted component. The DAF-2/DAF-16 insulin-signaling pathway has been shown to be involved in the starvation-induced arrest [3]. Further characterization revealed that C17ISO may play a critical role in activating post-embryonic development in *C. elegans* through a novel pathway that is independent of the DAF-2/DAF-16 food-sensing pathway [2]. CKI-1 is a cyclin-dependent kinase inhibitor that has been shown to be required for exit from the cell cycle and is likely a critical downstream target of DAF-16 with its expression level normally decreasing during L1 development [3],[4]. The L1 arrested larvae seen in the absence of C17ISO exhibit stable expression of CKI-1 [2], suggesting that C17ISO deprivation may contribute to the upregulation of CKI-1 through a previously uncharacterized, DAF-16-independent signaling pathway. Consistent with the critical regulatory role played by mmBCFAs in worms, we have found that mmBCFA homeostasis is maintained through a feedback regulatory mechanism involving SBP-1 [1],[2]. SBP-1, the *C. elegans* orthologue of mammalian SREBP-1c [5], may sense a deficiency in mmBCFA levels and respond by upregulating the transcription of monomethyl branched-chain fatty acid biosynthesis enzymes including ELO-5 and ACS-1, a long chain fatty acid acyl-CoA ligase.

Cell membranes are generally asymmetric in nature, containing higher levels of aminophospholipids on the cytosolic leaflet and exhibiting an enrichment of sphingolipids and choline-containing lipids on the extracellular/lumenal leaflet [6]. Alterations in cell surface properties due to the loss of membrane asymmetry are associated with various normal and pathological outcomes including apoptosis and platelet activation [7],[8]. However, with the exception of phosphatidylserine externalization during apoptosis, the precise mechanisms by which the asymmetric distribution of lipids across cellular membranes regulates membrane and lipid functions are not well understood. A subfamily of P-type ATPases, called aminophospholipid translocases or flippases, are proteins that are thought to contribute to this asymmetry in an ATP-dependent manner [9],[10]. The postulated function of flippases is to aid in the inward movement of phosphatidylserine (PS) and phosphatidylethanolamine (PE) from the extracellular or lumenal leaflet to the cytosolic leaflet of cellular membranes. To date, there have been very limited functional studies on this family of proteins in animals. In yeast, an aminophospholipid translocase, *DRS2*, has been shown to mediate the flipping of a fluorescent analog of PS in isolated late Golgi membranes [11]. *DRS2* mutants have been shown to have defects in protein trafficking at the *trans* Golgi network [12],[13], suggesting that membrane asymmetry may be important for intracellular transport events. In humans, mutations in *FIC1*, a putative aminophospholipid translocase, have been found in familial hereditary cholestasis [14]. However, the mechanism by which this mutation contributes to the pathology has yet to be elucidated. The *C. elegans* genome contains six predicted aminophospholipid translocases known as *tat-1* through *tat-6* [15],[16]. Recent work based on the expression pattern of reporter transgenes suggests that each of the TAT proteins may have distinct functions and four of them are not essential under regular growth conditions [17]. Mutations in *tat-2*, *tat-3* and *tat-4* genes were shown to enhance the sensitivity of *C. elegans* to cholesterol deficiency [17]. TAT-1 has been shown to be involved in the movement of phosphatidyl serine during apoptosis and regulate lysosome biogenesis and endocytosis [16],[18],[19]. However, the cellular and physiological functions of TAT proteins largely remain to be explored in animal cells.

Sphingolipids are structurally diverse lipids that can have equally diverse functions. In addition to altering membrane content, sphingolipid metabolites have also been implicated in intracellular and extracellular signaling (reviewed in [20]). The rate-limiting step in sphingolipid biosynthesis is the condensation of serine with fatty acid-CoA to generate 3-ketosphinganine, a precursor to the sphingoid base. This step is catalyzed by serine palmitoyltransferase (SPT) and as the name implies, the fatty acyl-CoA substrate is generally palmitoyl-CoA [21],[22]. In *C. elegans*, sphingoid bases of glucosylceramides and sphingomyelin have been shown to exclusively contain a branched-chain base constituent, perhaps produced from an iso-branched fatty acid [23]. This may suggest a potential link between the essential function of mmBCFAs and sphingolipids.

In this paper, we describe the identification and characterization of mutations in the *tat-2* P-type ATPase gene as suppressors of the L1 arrest caused by depleting the mmBCFA, C17ISO. We provide evidence that TAT-2 acts in intestinal cells to specifically antagonize mmBCFA activity in regulating post-embryonic growth, and such a function may be mediated by an mmBCFA-containing sphingolipid. This work provides a novel link between the critical regulatory function of a specific fatty acid and the maintenance of lipid bilayer asymmetry.

## Results

### Suppressor mutations define a gene that may act downstream of C17ISO to antagonize its function in regulating L1 growth

**Figure 1:**
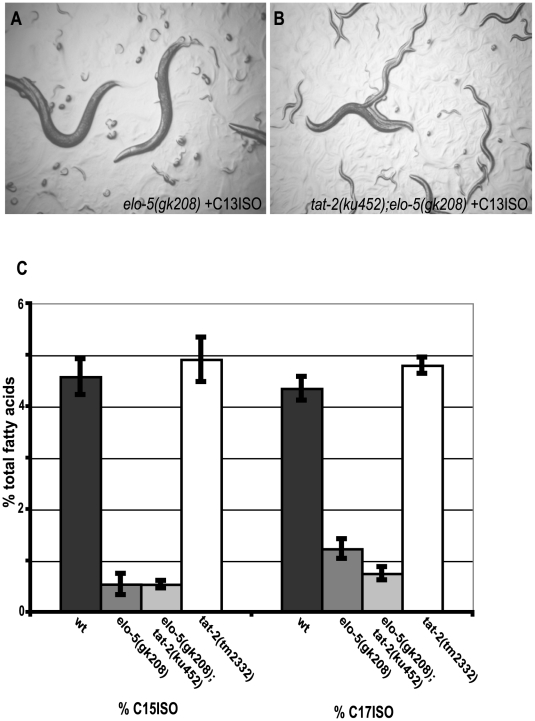
Suppressor mutations relieve L1 arrest of *elo-5(gk208)* animals without recovering the ability to synthesize C15ISO or C17ISO. (A) Image of a population of *elo-5(gk208)* animals grown from eggs on plates supplemented with C13ISO, showing P0 adults and arrested L1 larvae of the F1 generation on day 5. (B) Image of a population of *elo-5(gk208)*; *tat-2(ku452)* animals grown from eggs on plates supplemented with C13ISO, showing P0 adults and mixed stage larvae of the F1 generation on day 5. (C) Comparison of mmBCFA composition in four strains of indicated genotypes grown on plates supplemented with C13ISO. C15ISO and C17ISO are significantly decreased in *elo-5(gk208)* when compared to wild-type worms, and remain at a low levels in the strains in which either *tat-2(ku452)* or *tat-2(tm2332)* is coupled with the *elo-5* mutation. The *tat-2(tm2332)* mutation alone does not cause a decrease in C15ISO and C17ISO. Five replicates were done for each sample type. Data are expressed as a percentage of total fatty acids+/−SEM.

The *elo-5(gk208)* deletion mutant animals die in various larval stages and consequently do not reproduce. When the *elo-5(gk208)* deletion worms are supplemented with C15ISO or C17ISO, they grow to wild-type adults and can continue to grow and propagate in the presence of this supplementation [1]. However, upon supplementation with a short-chain mmBCFA, C13ISO, *elo-5(gk208)* worms grow to wild-type adults in the first generation and produce F1 progeny (Figure 1A). The progeny uniformly arrest in the first larval stage for several days before dying. To identify genes that may potentially act downstream of C17ISO to mediate this regulatory function, we screened for suppressor mutations that would allow C13ISO-supplemented *elo-5(gk208)* animals to grow past the L1 stage in the F1 generation (Materials and Methods). Among several suppressor mutations, three alleles were able to grow indefinitely in the presence of C13ISO supplementation (Figure 1B). Without any supplementation, these worms grew similarly to wild type in the first generation, but their progeny arrested and died as early larvae (Table 1), indicating that, although the suppressor mutations were able to overcome the L1 arrest in the absence of the longer chain mmBCFA, C17ISO, they were not able to bypass all mmBCFA functions.

**Table 1:** Mutations in *tat-2* suppress growth and developmental defects caused by depleting mmBCFA.

Genotype and supplementation	Generation Number	Stage(a)
Wild type (N2)	Indefinite	NA
*elo-5(RNAi)*	F1	L1
*tat-2(RNAi)*	Indefinite	NA
*elo-5(gk208)*	P0	L1–L4
*elo-5(gk208)*+C13ISO	F1	L1
*elo-5(gk208);tat-2(ku452)*	F1	L1–L3
*elo-5(gk208);tat-2(ku452)*+C13ISO	Indefinite	NA
*tat-2(tm2332)*	Indefinite	NA
*elo-5(gk208);tat-2(RNAi)*	F2	L1–L3
*elo-5(RNAi);tat-2(tm2332)*	>F4	L1–L3
*elo-5(gk208);tat-2(tm2332)* (b)	F2	L1
*elo-5(gk208);tat-2(tm2332)*+C13ISO	Indefinite	NA

All strains were grown from eggs isolated from gravid adults grown on OP50 supplemented with the mmBCFA-producing bacteria *S. maltophilia* and released by alkaline hypochlorite treatment. NA: not applicable; these strains did not arrest.

(a)Stage at which animals arrested growth.

(b)The *elo-5(gk208);tat-2(tm2332)* strain also contains the *unc-5(e53)* mutation.

Molecular cloning indicated that these three mutations are alleles of a single gene, *tat-2* (see below). We were able to conclude that these three mutations are likely loss of function mutations in the *tat-2* gene and that loss of *tat-2* gene function is responsible for the suppressor phenotype based on results from the following three sets of experiments. Firstly, we determined that *tat-2* RNAi phenocopies the mutations. When *tat-2(RNAi)*, but no mmBCFA supplementation, was applied, *elo-5(gk208)* deletion mutants grew to healthy adults in the first generation and produced progeny that were able to develop into egg-laying adults, with the next generation arresting in early larval stages (Table 1). Secondly, we acquired a 222-bp deletion mutation in *tat-2*, *tm2332*, from the National Bioresource Project in Japan, and showed that it can also suppress the L1 arrest phenotype of *elo-5(RNAi)*. When treated with *elo-5(RNAi)* in the absence of mmBCFA supplementation, *tat-2(tm2332)* mutant worms grew to wild-type adults in the first generation and continued to grow and propagate normally when transferred to fresh *elo-5* RNAi plates for multiple generations. The stronger suppressing effect seen in this assay likely reflects that fact that *elo-5(RNAi)* has a weaker mutant phenotype as that of *elo-5(gk208)* [2]. A similar effect was seen with another *tat-2* deletion mutation, *tm1773* (data not shown). Finally, we were able to rescue the *tat-2(ku449)* mutant phenotype (eliminating the suppressive role of *ku449*) by expressing the TAT-2 protein from a transgene under the control of the putative TAT-2 promoter as described below (Table 2). Therefore, combined with the finding that TAT-2 is not involved in the biosynthesis of mmBCFAs (see below), our results indicate that TAT-2 plays a negative role in C17ISO-mediated L1 growth regulation.

**Table 2:** Expression of *tat-2* cDNA in intestine, but not in three other tissues, rescues the mutant effect of *tat-2(ku449)*.

Promoter:Gene	% F1 arrested as eggs/L1
*tat-2*:GFP	0 (n = 297)
*tat-2*:*tat-2*	99.1 (n = 444)
*ges-1*:*tat-2*	93.1 (n = 562)
*sth-1*:*tat-2*	0 (n = 383)
*exc-5*:*tat-2*	0 (n = 172)
*col-10*:*tat-2*	1.2 (n = 643)

All constructs were injected into the *tat-2(ku449);elo-5(gk208)* background. Rescue was defined as the percentage of animals carrying the array (green) that had returned to the phenotype of *elo-5(gk208)* animals grown on C13ISO.

### *tat-2* encodes a putative P-type ATPase

**Figure 2:**
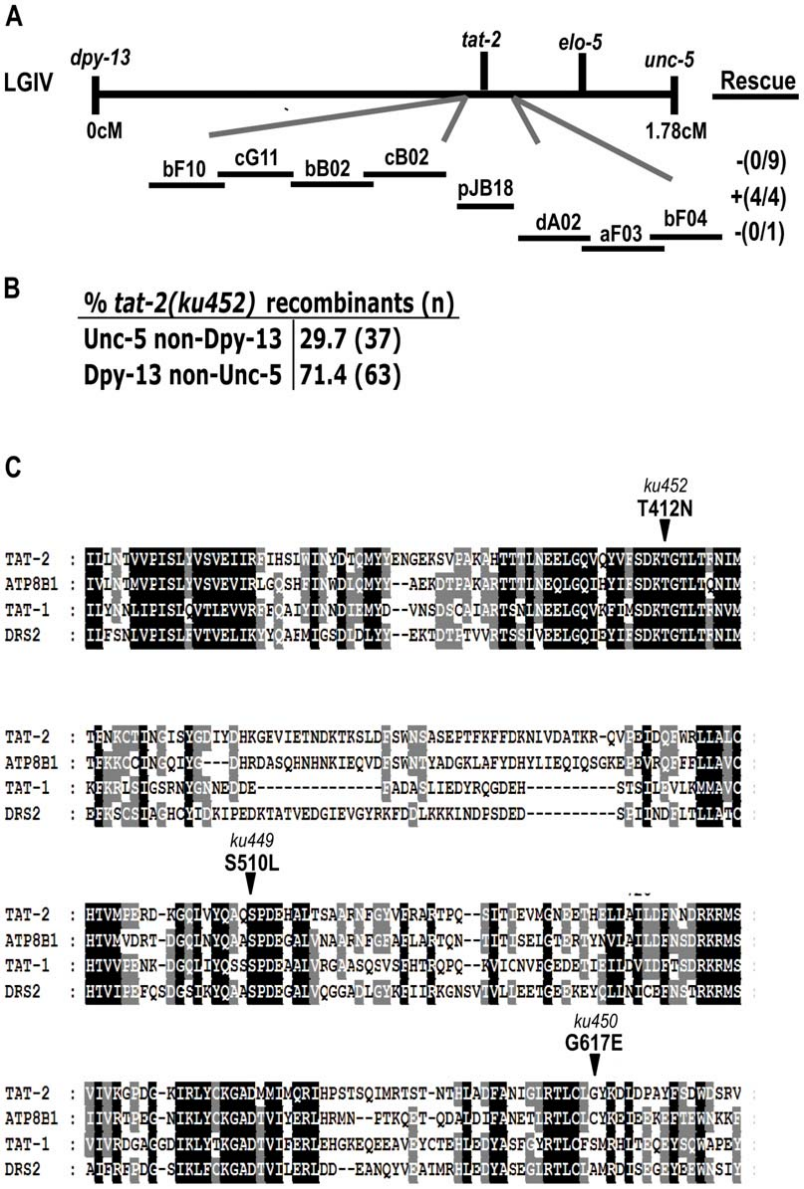
Positional cloning, protein alignment, and molecular lesions of *tat-2* defined by suppressors of the L1 arrest phenotype of *elo-5(lf)*. (A) Schematic representation of the region of *C. elegans* chromosome IV that indicates the position of the relevant genes and the phenotypic markers used for mapping. Also displayed are fosmids in the region, along with the area covered by the pJB18-rescuing construct. Numbers in parentheses represent the number of rescued lines out of the total number of lines generated. Fosmid abbreviations are short for: WRM0610bF10, WRM0640cG11, WRM0641bB02, WRM0631cB02, WRM0631dA02, WRM0617aF03, WRM0629bF04. (B) Shown in the table is the percentage of recombinants in the region that displayed the *tat-2(ku452)* suppressor phenotype. (C) Protein sequence alignment of a portion of *C. elegans tat-2* and *tat-1* (the P-type ATPase most closely related to *tat-2*), the yeast orthologue, DRS2 and the human orthologue of *tat-2*, ATP8B1. Residues shaded in black are identical among all proteins aligned. Residues shaded in gray are identical among two or more of the proteins aligned. Denoted with arrows and the corresponding allele names are the three amino acids in TAT-2 that are changed in the three suppressor mutants.

To identify the gene defined by the suppressor mutations, we mapped the mutations to the middle of chromosome IV using genetic and single nucleotide polymorphism markers (Materials and Methods; Figure 2A and 2B). Sequence analysis identified all three alleles as distinct molecular lesions in the *tat-2* gene: *ku452*, *ku449*, and *ku450* contained amino acid changes from Thr 412 to Asp, Ser 510 to Leu, and Gly 617 to Glu, respectively (Figure 2C). Upon examination of the *tat-2* cDNA, we found that it coded for an additional 92 amino acids not included in the protein predicted by Wormbase (data not shown).

The protein encoded by *tat-2* is a 1313-amino acid P-type ATPase that belongs to a family of proteins also known as flippases. Proteins with structural similarity to TAT-2 are found in yeast, mammals and *C. elegans* (Figure 2C). The protein encoded by *tat-2* shows conservation of domains that are characteristic of P-type ATPases as well as features that are specific to the Drs2p-subfamily of ATPases. Drs2p and other P-type ATPases are 10-pass transmembrane proteins with a cytoplasmic domain containing conserved residues that form an aspartyl-phosphate intermediate during ATP hydrolysis [24]. There are also two cytoplasmic sequences specifically conserved in the Drs2p family of ATPases [25]. The residue change in *tat-2(ku452)* (threonine 412 to asparagine) is two residues away from the catalytic aspartate and thus, may disrupt the catalytic function of the TAT-2 protein. This mutation may be of particular interest because an analogous mutation has been identified in patients diagnosed with familial cholestasis, in which this conserved threonine is changed to a methionine [26]. The *tat-2(ku449)* lesion (S510T) is in a cytoplasmic domain that is conserved in Drs2p-like ATPases, and the *tat-2(ku450)* lesion (G617E) affects a region that is not obviously conserved (Figure 2).

### *tat-2(lf)* does not alter mmBCFA biosynthesis or absorption, nor does it bypass all mmBCFA functions

To determine that the suppressor effect is not due to a recovery of C17ISO biosynthesis, we examined the fatty acid composition of these mutants by gas chromatography (GC). We found that the suppressor mutants grown in the presence of C13ISO did not recover the ability to synthesize mmBCFAs (Figure 1C). The GC analysis also detected statistically significant differences in the levels of two other fatty acids, stearic acid (C18:0; *P* = .016) and methyleneoctadecanoic acid (C19Delta; *P* = .0015) between wild-type, *elo-5(gk208)*, *elo-5(gk208)*; *tat-2(tm2332)* and *tat-2(tm2332)* animals. Because C19Delta was significantly increased in the *tat-2(tm2332)*; *elo-5(gk208)* mutants compared to *elo-5(gk208)* worms, we supplemented this fatty acid to the food given to *elo-5(gk208)* worms in the presence of C13ISO, but no rescue of the growth arrest of *elo-5(gk208)* mutants was observed (data not shown). Therefore, while these *tat-2* mutations allow mmBCFA-deficient animals to by-pass the L1 arrest, they do not do so by recovering the ability to synthesize mmBCFAs, nor by increasing the levels of other fatty acids.

Upon detailed examination of the *tat-2(tm2332)* single deletion mutant, we saw no obvious abnormalities, including normal fatty acid composition (data not shown; Supplemental Data; Figure 1). When we constructed the complete double deletion mutant, *tat-2(tm2332);elo-5(gk208)*, we found that the animals behaved similarly to the point mutants in that they can grow indefinitely in the presence of C13ISO. However, a small proportion of worms (<20%) grown without supplementation progressed an additional generation to F2 prior to the arrest, instead of arresting in the F1 generation as seen with the point mutants (Table 1). These data support the idea that the absence of *tat-2* can suppress some, but not all defects caused by mmBCFA deficiency. It also suggests that in the absence of *tat-2*, C13ISO (at a low concentration) can substitute for C17ISO in some post-embryonic developmental aspects. One possibility is that, in *tat-2(-)* animals, the absorption of C13ISO may be increased to achieve a higher cellular concentration of C13ISO. We tested this by feeding high amounts of supplemental C13ISO to *tat-2(-)* and N2 worms. By comparing the fatty acid composition of these worms, we determined that there was not a significant difference in absorption of C13ISO (data not shown).

### TAT-2 functions in the intestine to mediate C17ISO-deficiency-triggered growth arrest

To determine where *tat-2* is expressed in *C. elegans*, we generated a transcriptional GFP reporter that included approximately 3 kb of sequence upstream of the *tat-2* initiation codon. Consistent with the report by Lyssenko et al. [17], we also observed that *tat-2* is expressed primarily in the excretory cell, spermatheca and intestine (Figure S1 and Text S1). ELO-5 is also expressed in the intestine and several amphid neurons in the head [1]. Since fatty acids generated in the intestine are likely transported to various tissues of the body, it is possible that rescue of the *elo-5* deletion mutant phenotype depends on TAT-2-mediated effects on mmBCFAs in non-intestinal tissues. In order to address this, we first verified that the *tat-2* cDNA expressed under the control of the 3-kb promoter described above produces a functional protein by generating transgenic lines harboring a *tat-2*Prom::*tat-2* cDNA in the *tat-2(ku449);elo-5(gk208)* genetic background. The transgenic worms were assessed for the *elo-5(lf)* phenotype (second-generation larval arrest) in the presence of C13ISO and compared to transgenic *tat-2(ku449);elo-5(gk208)* worms expressing GFP under the control of the *tat-2* promoter. As shown in Table 2, 99.1% of the transgenic F1 worms expressing *tat-2* cDNA arrested at the L1 stage, whereas 0% of the negative control transgenic F1s arrested as L1s. This indicated that the *tat-2* cDNA expressed under the control of the 3-kb *tat-2* promoter is sufficient to rescue TAT-2 function. Next, we generated several constructs for the tissue-specific expression of the *tat-2* cDNA, and each of these constructs was injected into *tat-2(ku449)*; *elo-5(gk208)*. The transgenic lines were then examined for second-generation larval arrest in the presence of C13ISO and the results are summarized in Table 2. Expression of *tat-2* cDNA from the intestinal-specific *ges-1* promoter [27] was able to fully rescue the *elo-5* suppression phenotype of *tat-2(ku449);elo-5(gk208)*, however, *tat-2* driven by the spermathecal promoter, *sth-1* [28], the excretory cell promoter, *exc-5* [29], or the hypodermal-specific promoter *col-10* [30], was not able to restore the *elo-5* phenotype (Table 2). These results demonstrate that loss of functional TAT-2 in the intestine is necessary for suppression of *elo-5(gk208)*. Therefore, TAT-2 acts in the intestine for its function in C17ISO-defiency-triggered growth arrest.

### TAT-2, but not other TAT genes, is specifically involved in mmBCFA-mediated growth regulation

To test if the rescue of mmBCFA deficiency was a shared characteristic of depletion of other aminophospholipid translocases in *C. elegans*, we acquired deletion mutations for *tat-1(tm1034)*, *tat-3(tm1275)*, *tat-4(tm1801)* and *tat-6(ok1984)* and checked for rescue of the *elo-5(lf)* phenotype. Due to the poor viability of the *tat-5* deletion mutant and the penetrant embronyic lethal phenotype displayed by *rrf-3(-)* animals on *tat-5* RNAi, we treated *elo-5(gk208);rrf-3(ok629)* animals with *tat-5* RNAi and assayed rescue. None of these *tat* mutants rescued the *elo-5(lf)* phenotype (Table 3). In addition, we noted that *tat-4(tm1801)* and *tat-6(ok1984)* seemed to have a more severe phenotype in the presence of *elo-5(lf)*, resulting in death of the *tat-4* and *tat-6* mutant animals in the first generation (Table 3). While TAT-2 is the only TAT protein that plays a prominent negative role in mmBCFA-mediated growth regulatory functions, it is possible that TAT-4 and TAT-6 play some positive roles in mmBCFA-involved functions.

**Table 3:** Effects of mutating five other P-type ATPase in *C. elegans* on the *elo-5(lf)* growth arrest phenotype.

Genotype	% Larvae Past L1 in 2^nd^ Generation (n)(a)
*elo-5(gk208)*+C13ISO	0 (40)
*elo-5(gk208);tat-2(ku452)*+C13ISO	99 (44)
*elo-5(gk208);tat-1(tm2232)*+C13ISO	0.3 (47)
*elo-5(RNAi)*	0 (20)
*elo-5(RNAi)*; *tat-2(tm2332)*	100 (40)
*elo-5(RNAi)*; *tat-3(tm1275)*	0 (20)
*elo-5(RNAi)*; *tat-4(tm1801)*	0(b) (18)
*elo-5(RNAi)*; *tat-6(ok1984)*	0(c) (46)
**Genotype**	**Phenotype**
*rrf-3(ok629)*; *tat-5(RNAi)*	embryonic lethal (14)
*Rrf-3(ok629)*; *elo-5(gk208)*; *tat-5(RNAi)*	larval lethal(d) (14)

Wild-type and mutant strains for the RNAi feeding experiments were grown from eggs on *elo-5*(RNAi) and control HT115 plates. Rescue (number of larva past L1 in the second generation) was assayed on the day 6–7. For the *elo-5(gk208)* double mutant experiments, worms were grown from eggs on OP50 bacteria supplemented with 1 mM C13ISO. The numbers of larva past L1 larval stage were counted on day 6–7. In the RNAi feeding experiments with *tat-5* RNAi, rescue of the *elo-5(gk208)* larval lethal phenotype in the first generation was assayed.

(a)n = number of plates.

(b)no eggs laid by the P0, suggesting a synthetic sterile effect.

(c)synthetic lethal in the first generation, *tat-6(ok1984)* on HT115 alone is 50% embryonic lethal.

(d)in the first generation.

We then wanted to test if *tat-2(lf)* can also affect the growth defects caused by general fatty acid depletion. We grew *tat-2(tm2332)* and wild-type control animals on bacteria expressing RNAi against either *fasn-1*, the *C. elegans* fatty acid synthase orthologue or *fat-2*, a Δ12 fatty acid desaturase involved in the normal formation of polyunsaturated fatty acids [31],[32]. *tat-2(tm2332)* exhibited no rescue of the lethality caused by *fasn-1(RNAi)* or *fat-2(RNAi)* (data not shown).

### *tat-2(lf)* suppresses the lethality caused by disrupting sphingolipid biosynthesis

As mentioned earlier, sphingolipids in *C. elegans* have been shown to contain monomethyl branched-chain sphingoid bases [23]. We reasoned that *tat-2(lf)* may suppress the mmBCFA growth defect through suppression of the activity of more complex lipids that are formed from mmBCFAs, such as sphingolipids. We explored the idea that TAT-2 may act through its postulated flippase activity to modulate the activity of one or more sphingolipids localized in the membrane.

**Figure 3:**
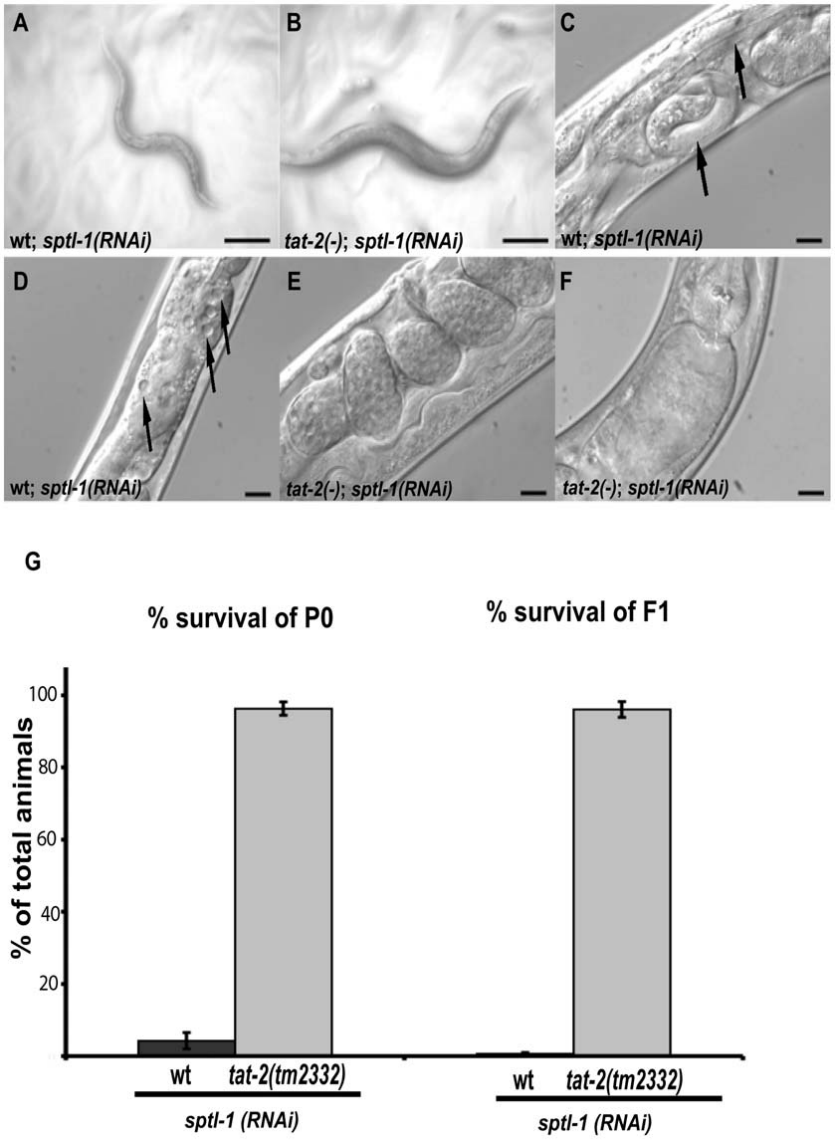
*tat-2(lf)* mutations suppress phenotypes caused by disrupting sphingolipid biosynthesis. (A–F) Images under Nomarski optics of wild-type or *tat-2(tm2332)* worms grown from eggs treated with *sptl-1*(RNAi). Images taken on day 5 of treatment. (A) The *sptl-1(RNAi)* treated wild-type adults shown are egg-laying defective which results in an uncoordinated morphology. The progeny are not visible on the plate. (B) The *tat-2(tm2332)*; *sptl-1*(RNAi) adults displayed normal movement and gross morphology. Eggs and progeny are visible. (C) An egg-laying defective worm with a larva (black arrows) hatched inside the mother. (D) Representative adult worm with vacuoles in the body and intestinal lumen (black arrows). (E) A non-egg-laying defective *tat-2(tm2332)* worm. (F) Representative *tat-2(tm2332)* worm with wild-type morphology. (G) Comparison of percent survival of the P0 generation and F1 generation after 10 days on *sptl-1*(RNAi)+/−SEM. n = 3 experiments with clonal analysis of 20 worms each; *P*<.001. Scale bars: (A–B) ∼100 µM; (C–F) ∼10 µM.

We examined the effects of depleting the *C. elegans* homolog (*sptl-1*) of one of the subunits (*SPTLC1*) of the mammalian serine palmitoyl-CoA transferase complex. We found that *sptl-1(RNAi)* results in 95.8% lethality in 10 days (Figure 3G). These animals also displayed an uncoordinated phenotype resulting from the dead larva within the mother and vacuolization and degradation of the intestine (Figure 3A and 3C). These results suggest that sphingolipids are essential for *C. elegans* survival and development. Surprisingly, when *tat-2(tm2332)* animals were grown in the *sptl-1(RNAi)* background, 96.3% were alive on day 10 (Figure 3G). The animals were egg-laying competent and most embryos developed normally (Figure 3E). A few embryos (>10 per worm) completed embryonic development inside the eggshell within the mother, but did not hatch out of the eggshell (data not shown). *tat-2(-);sptl-1(RNAi)* laid approximately 45% of the number of eggs compared to control *tat-2(-)* worms on the mock RNAi plates, and 96% of the resulting larvae were alive on day 10 (Figure 3G). The F1 larvae remain alive in various larval stages as long as 2 months but did not continue to propagate. In addition, no vacuoles were observed in *tat-2(tm2332)*; *sptl-1(RNAi)* worms (Figure 3F). This suppression is not likely due to a reduced efficiency of the RNAi treatment in the *tat-2* mutant, since no obvious reduction in RNAi efficiency was observed when the *tat-2* mutant was treated by RNAi of several other metabolic genes such as *fasn-1*, *spb-1 and acs-1* (data not shown). These results demonstrate that *tat-*2 deficiency not only rescues the growth defects caused by depletion of C17ISO, but can also partially rescue defects caused by depletion of sphingolipids.

Since cholesterol and sphingolipids reside together in lipid rafts, we tested *tat-2* mutants for the ability to suppress the cholesterol-depleted phenotype. We found no rescue of the growth arrest phenotype caused by cholesterol-depletion in *tat-2(tm2332)* worms as compared with wild-type worms (data not shown; [33]). We also sought to determine if *tat-2(tm2332)* would be able to rescue general lipid misregulation phenotypes. When we knocked down expression of the *C. elegans* homolog of SREBP gene, *C. elegans sbp-1*, which is thought to affect global lipid homeostasis [5], *tat-2(tm2332)* mutants showed no differences as compared to control worms under the same conditions (data not shown). Finally, we tested the ability of *tat-2(-)* to alter the phenotype of a deletion mutant in a *C. elegans* orthologue of phosphatidylserine synthase, *pssy-2(tm1955)*, an enzyme that converts phosphatidylcholine to phosphatidylserine. We found that the depletion of TAT-2 did not change the sterility phenotype of this mutant (data not shown).

### Glycosphingolipids contain C15ISO and C17ISO-branched fatty acid chains

**Figure 4:**
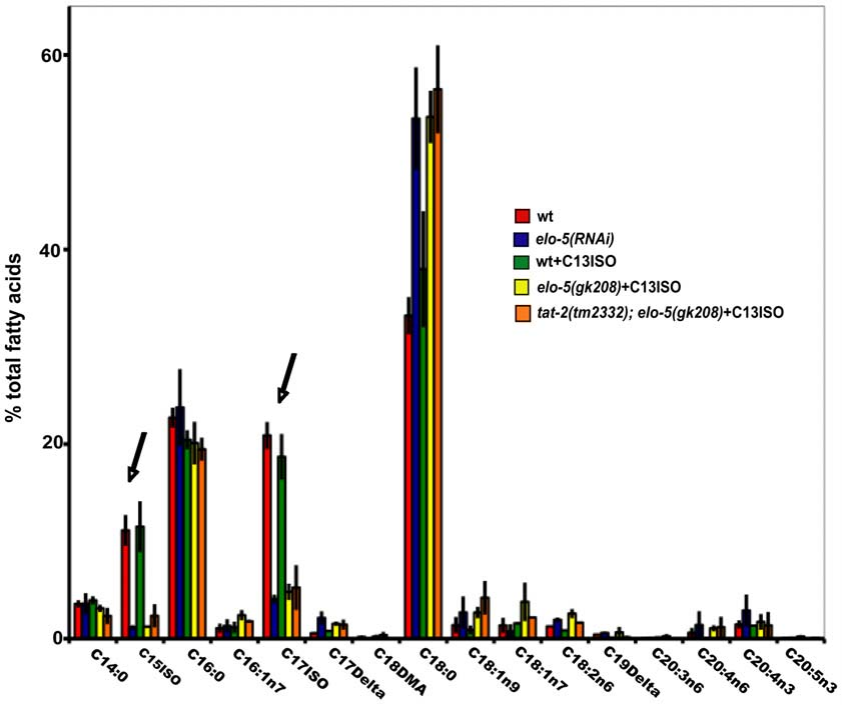
Glucosylceramides from *elo-5(lf)* worms lack mmBCFA constituents. Comparison of the fatty acid chains of low complexity glucosylceramides in 5 different strains as indicated. C15ISO and C17ISO are significantly decreased in the *elo-5*(RNAi), *elo-5(gk208)*+C13ISO and *tat-2(tm2332);elo-5(gk208)*+C13ISO animals when compared to the wild-type and wild-type +C13ISO (black arrows). The *tat-2(tm2332);elo-5(gk208)* strain also contains the *unc-5(e53)* allele. Data are expressed as percentage of total fatty acids+/−SEM. The data were collected from at least three replicates for each strain, except *tat-2(tm2332)*; *elo-5(gk208)*+C13ISO, which had two replicates; *P*<.001.

The rescue of both sphingolipid and C17ISO depletion by *tat-2* mutants led us to ask whether there were sphingolipids in *elo-5*-depleted worms and if so, what the fatty acid constituents of those sphingolipids were. Due to the difficulty of analyzing every type of sphingolipid in *C. elegans* in various mutant backgrounds, we chose to isolate glycosphingolipids in synchronized populations of mutant versus wild-type worms and separate them using thin layer chromatography (TLC) in the manner described in Griffitts et al., [34]. The bands representing ceramide-based sphingolipids with one to four saccharide molecules (as inferred by reference standards) were scraped from the TLC plates and the fatty acid side chains were extracted and analyzed by gas chromatography. The results are displayed in Figure 4. Analysis of wild-type control worms indicated that C15ISO and C17ISO represent ∼20–30% of the fatty acid chain composition in the glycosphingolipids obtained in this assay. In the *elo-5*-depleted worms, the fatty acid composition in the TLC scrape revealed the combined C15ISO and C17ISO proportions were down to ∼5%. The *elo-5(lf)* and wild-type worms supplemented with C13ISO showed similar results. In addition, we noted that the *tat-2(lf);elo-5(lf)* animals supplemented with C13ISO had similar results to the *elo-5(lf)* animals (Figure 4). This likely rules out the possibility that *tat-2(-)* is somehow rescuing the growth defect by altering the incorporation of mmBCFAs into the fatty acid side-chains of sphingolipids. When the relative amount of glycosphingolipids in the wild-type worms was compared with that in the *elo-5(RNAi)* worms, they were found to be similar (data not shown). Interestingly, *elo-5(RNAi)* worms incorporate other fatty acids into glycosphingolipids in order to compensate for the missing mmBCFAs, with a trend towards a significant increase in stearic acid (C18:0). We postulate that the substituted glycosphingolipids are unable to carry out the essential functions of mmBCFA-containing sphingolipids required for post-embryonic growth.

## Discussion

### Role of TAT-2 in mmBCFA-mediated growth regulation

**Figure 5:**
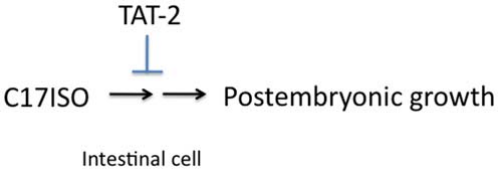
Proposed functional relationship between C17ISO and TAT-2 in promoting post-embryonic growth and development. C17ISO is required for post-embryonic growth and development. TAT-2 antagonizes this function in intestinal cells, possibly by affecting the process of generating an effective mmBCFA-containing lipid molecule in proper cellular locations.

Little is known about the functions of most of the P-type ATPases in animals. In this report, we identified a novel physiological role for a P-type ATPase in a specific lipid-mediated developmental function. In our previous studies, we showed that depletion of long monomethyl branched-chain fatty acids by mutating the *elo-5* gene causes *C. elegans* to robustly arrest their post-embryonic growth. We show here that loss-of-function mutations in the putative aminophospholipid translocase TAT-2 are able to overcome this arrest, indicating a link between TAT-2 function at cell membranes and C17ISO-mediated growth regulation. A straightforward interpretation of the genetic data is that TAT-2 antagonizes C17ISO function in promoting post-embryonic growth (Figure 5). Given what is known about this family of P-type ATPases, we speculate that TAT-2 may regulate the function of a lipid of more complex structure that mediates the C17ISO functions. TAT-2 could function to direct the localization of this lipid through its potential lipid translocase activity or through its potential role in secretory and/or endocytic trafficking.

Our work presented in this paper also suggests that such a lipid affected by TAT-2 function may be a sphingolipid. We have found that *tat-2* mutations also partially suppress the developmental defects caused by RNAi of a subunit of the serine-palmitoyl transferase complex that is required for sphingolipid biosynthesis. We further determined that ceramide-based glycosphingolipids contain a significant proportion of C15ISO and C17ISO in their fatty acid side chains and that these are depleted in an *elo-5(lf)* background. Although it is currently corollary evidence, these results strongly implicate a functional linkage between mmBCFAs and sphingolipids.

In a recent report, mutations in *tat-2*, *tat-3* and *tat-4* genes were shown to enhance the sensitivity of *C. elgans* to cholesterol deficiency, implicating the roles of these P-type ATPases in cholesterol metabolism [17]. These roles appear to be distinct from the *tat-2* function in C17ISO-mediated growth regulation for two reasons. First, regarding the effects on the animal growth, the enhancement of the defects caused by cholesterol deficiency is the opposite to the rescue of the defects caused by C17ISO depletion or by disrupting sphingolipid biosynthesis. Second, the *tat-2* mutant effect on C17ISO function is highly specific to this particular P-type ATPase; none of the other *tat* genes has any observable effects (Table 3).

Disruption of mmBCFA biosynthesis causes multiple developmental defects that include, but are not limited to L1 growth arrest [1]. There is a clear functional difference between the shorter mmBCFA C13ISO and the long chain mmBCFA C17ISO [2]. Low levels of exogenous C13ISO (1 mM) can overcome almost all developmental defects caused by depleting endogenous mmBCFAs in *elo-5* mutants except the L1 growth arrest that can be overcome by feeding a low level of dietary C17ISO or a high level of C13ISO (10 mM). Our data in this study indicate that *tat-2* mutations can suppress the L1 arrest phenotype but not bypass the requirement of mmBCFA for some other developmental functions. In our typical L1 arrest assay, the *elo-5(lf)* mutants were grown on plates supplemented with a low level of C13ISO. These results indicate that C17ISO, not C13ISO, plays the major role in mediating development past the first larval stage in normal worms, and that C13ISO, at a very high concentration, can function as C17ISO for the growth regulatory function. The latter raises the possibility that the *tat-2* mutations may suppress the growth arrest by dramatically changing the dynamics or efficiency of C13ISO. For example, *tat-2* mutant worms may be able to absorb a higher level of exogenously provided mmBCFAs, leading to a higher internal C13ISO concentration. Alternatively, in the absence of TAT-2, the subcellular distribution of C13ISO may be altered through a novel mechanism in such a way that allows the molecule to function as effectively as C17ISO.

Several lines of evidence argue that the above scenarios are unlikely. First, under conditions without any mmBCFA supplement, *elo-5(-);tat-2(-)* mutants can typically bypass the L1 arrest and grow another generation before the animals arrest at various larval stages due to other developmental defects (Table 1). In addition, when *elo-5(RNAi)* is applied to wild-type worms, L1 arrest is observed for the F1 animals. However, when *elo-5(RNAi)* is applied to *tat-2(-)* mutants without any mmBCFA supplementation, the animals can actually propagate multiple generations without developmental arrest (Table 1). These results suggest that the *tat-2* mutations are able to overcome the L1 arrest from depleting C17ISO without the presence of C13ISO. Second, we found that internal C13ISO levels were not significantly different between *tat-2* and wild-type worms, arguing against the possibility that a *tat-2* mutation enhances the absorption of C13ISO into animal cells. Third, the C15ISO and C17ISO levels are similar in the *elo-5;tat-2* double mutants when compared to the *elo-5* single mutant, inconsistent with the idea that a *tat-2* mutation can increase the stability of mmBCFAs. Finally, if the C17ISO function in post-embryonic growth is mediated by a glycosphingolipid as we proposed above, the fact that *tat-2* mutations can also suppress the defects in *sptl-1(RNAi)* worms does not appear to be consistent with the effect of the *tat-2* mutations altering the subcellular distribution and effectiveness of C13ISO.

### mmBCFA and TAT-2 function in the intestine to regulate post-embryonic growth

Our previous work indicated that the mmBCFA C17ISO can move from the intestinal cells of adult worms into their eggs [2]. Therefore, mmBCFA produced in a given tissue can potentially move to other tissues, making it difficult to determine in which tissue the mmBCFA is promoting post-embryonic growth. In this study, by expressing the TAT-2 protein behind tissue-specific promoters, we were able to show that expression of TAT-2 in intestinal cells is sufficient and likely necessary for TAT-2 to execute its role in mmBCFA-mediated regulatory function (Table 2). The latter statement is supported by the data that the expression of TAT-2 in three other tissues, including the major hypodermal tissue, could not rescue the mutant phenotype. Consistent with this, the mmBCFA biosynthesis enzymes ELO-5, ELO-6 and ACS-1 are all expressed at high levels in intestinal cells. These results suggest that the post-embryonic growth regulatory function of mmBCFAs and TAT-2 primarily occurs in intestinal cells, where exogenic mmBCFAs would have been first absorbed during feeding. However, because depleting C17ISO causes arrest of all post-embryonic tissues including muscle cells and hypodermal cells [2], a growth inhibitory signal such as an increase in *cki-1* expression that may be triggered by C17ISO deficiency may be able to spread from intestinal cells to neighboring cells. The nature of this high-order lipid molecule and how this molecule executes this signaling process are currently not known. Additional genetic screening and biochemical analyses are being carried out to search for the answers.

## Materials and Methods

### General *C. elegans* maintenance and strains

All strains were maintained at 20 °C on OP50 bacteria on nematode growth media (NGM) according to standard protocol [35] unless RNAi treatment was performed. The wild-type strain was Bristol strain, N2. Mutant strains used were *elo-5(gk208)*IV, *tat-2(ku449)*IV, *tat-2(ku450)*IV, *tat-2(ku452)*IV, *tat-2(tm2332)*IV, *tat-2(1773)*IV, *tat-1(tm1034)*III, *tat-3(tm1275)*III, *tat-4(tm1801)*II, *tat-6(ok1984)*V, *rrf-3(ok629)*II, *dpy-13(e184)*IV and *unc-5(e53)*IV. The *elo-5(gk208)*IV, *tat-6(ok1984)*V, *rrf-3(ok629)*II, *dpy-13(e184)*IV and *unc-5(e53)*IV strains were obtained from the *C. elegans* Gene Knockout Consortium. The *tat-2(tm2332)*IV, *tat-2(1773)*IV, *tat-1(tm1034)*III, *tat-3(tm1275)*III, *tat-4(tm1801)*II, *pssy-2(tm1955)*III deletion strains were obtained from the National Bioresource Project in Japan. The CB4856 Hawaiian strain was used in SNP mapping procedures.

The *elo-5(gk208)*IV strain and suppressor and double mutant strains with the *elo-5(gk208)*IV allele were maintained on OP50 bacteria supplemented with the mmBCFA-producing *S. maltophilia* bacteria [1].

### *elo-5(lf)* suppressor screen

*elo-5(gk208)* L4-staged P0 worms grown in the presence of 2 mM C17ISO were mutagenized with EMS. Mutagenized F1 worms were grown to gravid adulthood on 2 mM C17ISO-supplemented plates and F2 eggs released by alkaline hypochlorite treatment were plated on 1 mM C13ISO-supplemented plates. Suppressor candidates were determined by the ability of their progeny to grow past the L1-stage. From ∼8000 haploid genomes, five suppressor mutants were isolated. Three *tat-2* alleles, *ku449*, *ku450*, *ku452*, were isolated in separate rounds of screening.

### Phenotypic scoring

For all survival and rescue experiments, strains were maintained on plates containing *S. maltophilia* to provide an exogenous source of mmBCFAs. In order to score for *elo-5* growth arrest phenotypes, worms were collected from *S. maltophilia* containing plates and subjected to alkaline hypchlorite treatment to eliminate contaminating *S. maltophilia* and eggs were collected. Clean eggs were then spotted onto AMP plates spotted with HT115 transformed with empty vector (pPD 129.36) without mmBCFA supplements or AMP plates spotted with HT115 transformed with empty vector to which indicated concentrations of C13ISO were added from a 10 mM stock in DMSO. Following several days of growth, worms were scored for their developmental stage. HT115, when transformed with pPD 129.36, was used in these experiments because of its resistance to ampicilin, which aids in preventing the growth of contaminating bacteria that can interfere with accurate rescue scoring. Phenotypes on standard OP50 were identical.

In the *sptl-1(RNAi)* experiments, gravid wild-type or *tat-2(tm2332)* adults were bleached and eggs were spotted on bacteria expressing *sptl-1(RNAi)*. After 2 days of development, 20 single worms were cloned to individual *sptl-1(RNAi)* plates and survival of the P0 and F1 generation was quantified as a percent of the total number of animals scored. This was repeated 3 times, in parallel for wild-type and *tat-2(tm2332)* mutant worms. An unpaired Student's t-test was used to compare the values.

### Analysis of fatty acid composition by gas chromatography

Lipid extraction, fatty acid methyl ester preparation and gas chromatography were performed in the manner described [1],[2],[36]. Worms grown from eggs isolated by bleaching were spotted on plates with: 1) HT115 (transformed with pPD129.36), 2) HT115 supplemented with C13ISO where final concentration of the fatty acid in the bacteria culture was equal to 1 mM, or 3) HT115 transformed with pPD129.36 vector expressing double-stranded RNA against the indicated genes. Adults and their L1 progeny were collected with water, washed and frozen until use for lipid extraction. For each type of sample, five replicates were assayed.

For analysis of the fatty acid chains in glycosphingolipids, glycosphingolipids were first isolated from worm extracts and separated by TLC as described below. Bands representing glycosphingolipids were scraped from the silica TLC plates. Extraction, methyl ester preparation and gas chromatography of fatty acids from TLC-fractionated lipids were performed as outlined above. In these experiments, three replicates were done for each sample type, except where noted. For both analyses, average, standard deviation and standard error were calculated using the Excel program. Statistical significance was determined using the single-factor ANOVA method.

### Molecular cloning of *tat-2*

A SNP mapping method [37] was used to map *tat-2(ku452)* near the middle of chromosome IV. Due to the proximity of *tat-2(ku452)* to the *elo-5(gk208)* mutation, which was necessary to have in the background to observe the suppressor phenotype, the mapping strategy was switched to traditional three-point mapping, narrowing the region containing *ku452* to between the markers *dpy-13* and *unc-5*. Recombinants were generated by crossing *ku452* to a *dpy-13(e184)*; *elo-5(gk208)*; *unc-5(e53)* strain. F2 Unc non-Dpy and Dpy non-Unc animals were isolated, homozygosed and scored for the presence of *ku452* suppressor phenotype. Of these recombinants, 11/23 Unc non-Dpy and 45/63 Dpy non-Unc recombinants retained the *ku452* mutation. Pools of four fosmids (15 ng/µL) along with *sur-5::GFP* (90 ng/µL) [38] were co-injected into *ku452* worms and examined for rescue. Due to the incomplete fosmid coverage of the *tat-2* gene, no fosmid pools rescued. Therefore, pJB18 (see below) was injected and rescue was observed. The coding region of *tat-2* was PCR-amplified from *ku449*, *ku450* and *ku452* mutants and sequenced to identify the molecular legions.

### RNAi feeding

HT115 bacteria expressing double-stranded RNA from the indicated genes was seeded onto NGM agar plates with IPTG and ampicilin for feeding [39]. Bacterial strains were obtained from the *C. elegans* genome-wide RNAi feeding library (Geneservice). Eggs isolated from the indicated *C elegans* strains by hypochlorite treatment were allowed to develop at 20°C with the RNAi feeding strains as their food source. Controls were fed with HT115 transformed with an empty pPD129.36 plasmid.

### Expression, rescue, and heat shock constructs

The *tat-2* transcriptional reporter construct, pJB10, was generated by PCR amplification of 3 kb upstream of the *tat-2* initiation codon from N2 genomic DNA using the following primers: For 5′-AAGGATCCTTTCCATGACTCACGCTG-3′, Rev 5′-AACCCGGGCCCTCCGCCACCTCCTTT-3′. The PCR product was ethanol precipitated, digested with BamHI and SmaI and ligated into the GFP vector pPD95.69 (generously provided by the Fire lab). The resulting construct, pJB10, was co-injected with pBluescript into the following strains: N2, *elo-5(gk208)*, *elo-5(gk208);ku452*, and *tat-2(tm2332)* to obtain transgenic lines. Multiple lines were generated in each strain to verify the expression. A construct expressing *tat-2* cDNA under the native 3 kb *tat-2* promoter was generated by first cloning the promoter into pPD49.26 using the same primers and procedure described above for pJB10. The resulting construct, pJB14, was subsequently used to subclone the *tat-2* cDNA. A first strand cDNA synthesis reaction was carried out using a gene-specific *tat-2* primer and RNA isolated from N2 worms as template. The *tat-2* cDNA was then PCR amplified from the first strand reaction using the following primers: For 5′-CACACCCGGGATGTTCAGTTGGTTGCCATG-3′, Rev 5′-AAACCCGGGTTAAAGGCGAGTGATTACGTCTG-3′. The PCR product was ligated into pJB14 as a SmaI fragment to yield pJB18. To generate tissue-specific constructs *tat-2* cDNA was amplified from the first strand reaction described above using primers: For 5′-CACAGGATCCATGTTCAGTTGGTTGCCATG-3′, Rev 5′-AAACCCGGGTTAAAGGCGAGTGATTACGTCTG-3′. The PCR product was ligated into pPD49.26 as a BamHI/SmaI fragment to generate pJB15. Tissue-specific promoters were PCR amplified as follows: 1.8 kb upstream of the *ges-1* start codon, 2.2 kb upstream of the *exc-5* start codon and 3.4 kb upstream of the *sth-1* start codon. Each PCR product was purified, digested and subcloned separately into pJB15 upstream of the *tat-2* cDNA start codon. For the heat shock construct, pES11, the *tat-2* cDNA generated above was cloned into the heat shock promoter vector, pPD49.83 as a BamHI/SmaI fragment. pES11 was co-injected with pBluescript and with a *sur5::GFP* marker (Yochem et al. 1998) construct into N2 worms.

### Microscopy

Analysis of GFP expression and phenotypic abnormalities were conducted with Nomarski optics using a Zeiss Axioplan2 microscope and a C4742-95 CCD camera. Plate phenotypes were observed through a Leica MZ16F dissecting microscope and pictures were taken with a Canon Powershot A620 digital camera with a Canon Scopetronix Maxview Plus adapter.

### Glycosphingolipid extraction and thin-layer chromatography

Lower phase lipids were isolated and resolved via TLC in the manner described [34] from the indicated strains. In short, a 500–800 µL pellet of packed worms was used as starting material. The lower phase extracts were re-suspended in 500–800 µL volume of 1∶1 chloroform∶methanol. HPTLC plate (Supelco, Bellefonte, PA) lanes were loaded with 50 µL volumes of individual samples. Seven lanes were done for each sample type. The plates were developed in a TLC chamber containing 45∶18∶3 chloroform∶methanol∶water. Glycosphingolipids were resolved with an orcinol/sulfuric acid stain, followed by heating for 120 °C for 10 minutes. As mentioned in the results, a neutral glycosphingolipid standard was used for comparison. After cooling, the brownish-red bands corresponding to glycolipids were scraped from the plate and the bands from the seven lanes were pooled to obtain adequate material for fatty acid extraction and GC analysis.

## Supporting Information

Figure S1 TAT-2 is primarily expressed in the intestine, spermatheca and excretory cell (A, D, G, J,) DIC images, (B, E, H, K) fluorescence images and (C, F, I, L) merged images of various staged animals expressing the tat-2 Prom::GFP transgene. (A–C) An early staged larvae expressing GFP in the excretory cell (white arrow), pharyngeal muscle cells (black arrows), as well as strongly in the intestine. The result is mostly consistent with a previous analysis [17]. (D–F) Shown is the spermathecal expression in an adult animal. (G–I) The expression in the uterus of an adult animal (white arrow points to the uterus surrounding a developing embryo) is shown. (J–L) tat-2Prom::GFP is expressed in an amphid sheath cell (white arrow) extending along the head of an L1 larvae.
(6.50 MB TIF)

Text S1 Supplemental data.
(0.03 MB DOC)
