# Supplementary figures and images for: P-Type ATPase TAT-2 Negatively Regulates Monomethyl Branched-Chain Fatty Acid Mediated Function in Post-Embryonic Growth and Development in C. elegans
Source: PLoS Genet. 2009 Aug 7;5(8):e1000589. doi: 10.1371/journal.pgen.1000589 (PMC2716530; doi:10.1371/journal.pgen.1000589)

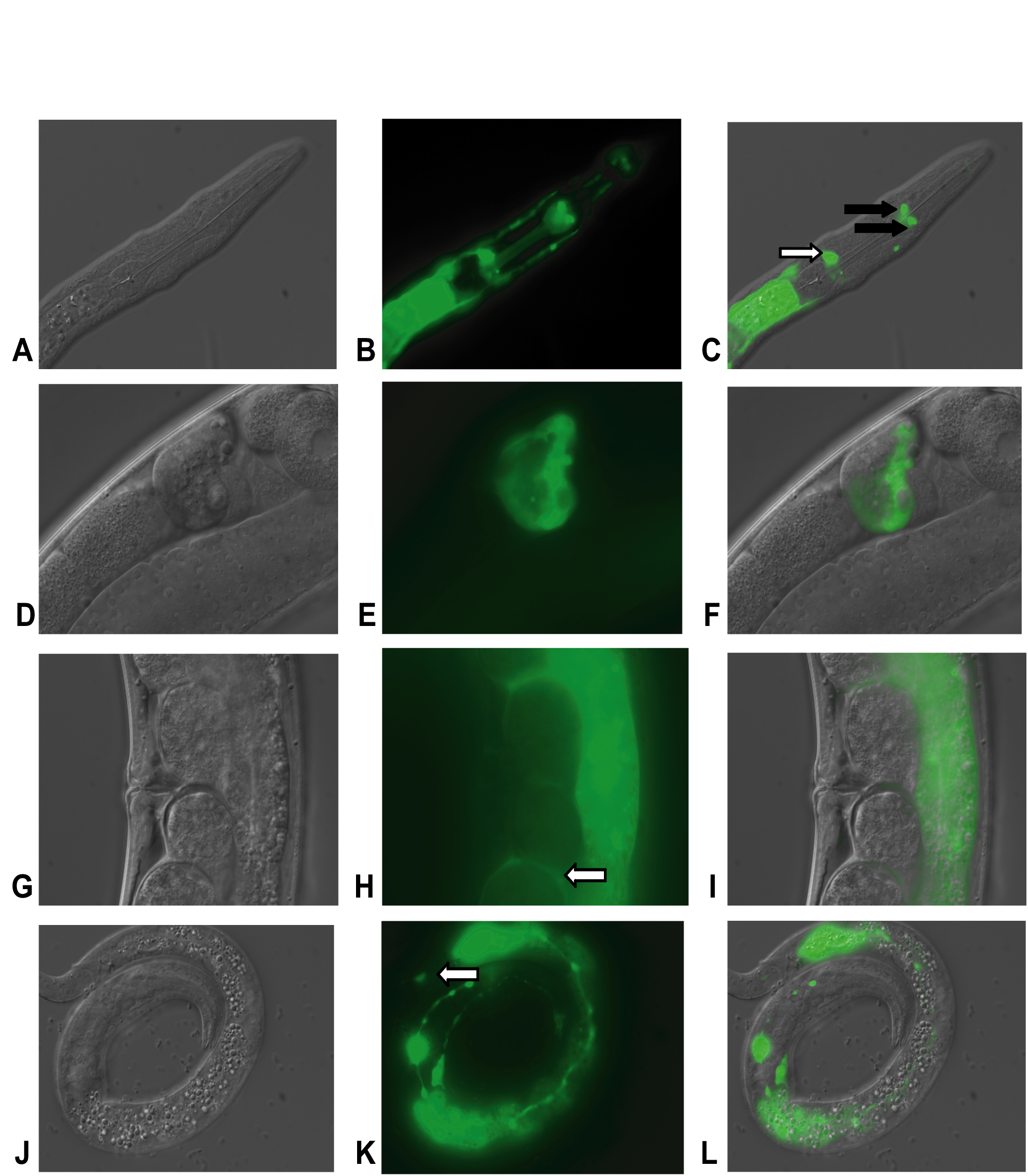

Supplement: Figure S1 — TAT-2 is primarily expressed in the intestine, spermatheca and excretory cell (A, D, G, J,) DIC images, (B, E, H, K) fluorescence images and (C, F, I, L) merged images of various staged animals expressing the tat-2 Prom::GFP transgene. (A–C) An early staged larvae expressing GFP in the excretory cell (white arrow), pharyngeal muscle cells (black arrows), as well as strongly in the intestine. The result is mostly consistent with a previous analysis [17]. (D–F) Shown is the spermathecal expression in an adult animal. (G–I) The expression in the uterus of an adult animal (white arrow points to the uterus surrounding a developing embryo) is shown. (J–L) tat-2Prom::GFP is expressed in an amphid sheath cell (white arrow) extending along the head of an L1 larvae. (6.50 MB TIF) [file pgen.1000589.s001.tif]
