# Supplementary material for: P-Type ATPase TAT-2 Negatively Regulates Monomethyl Branched-Chain Fatty Acid Mediated Function in Post-Embryonic Growth and Development in C. elegans
Source: PLoS Genet. 2009 Aug 7;5(8):e1000589. doi: 10.1371/journal.pgen.1000589 (PMC2716530; doi:10.1371/journal.pgen.1000589)
Supplement: Text S1 — Supplemental data. (0.03 MB DOC) [file pgen.1000589.s002.doc]

Supplemental Data

TAT-2 is expressed in the intestine, spermatheca and excretory canal

ELO-5 is expressed in the intestine and several amphid neurons in the head [1]. We generated a transcriptional GFP reporter that included approximately 3,000 base pairs of sequence upstream of the *tat-2* initiation codon. Functional data described in Table 2 demonstrated that this promoter region was able to drive the expression of a rescuing *tat-2* cDNA construct. Fluorescence of the expression of the transgene was first detected in the 1.5-fold embryo and continued through adulthood. The predominant expression pattern, shown in Figure S1, included the intestine, the excretory canal cell and from the L4 stage through adulthood, the spermatheca. We also detected expression in the uterus and various cells in the head including an amphid sheath cell and several pharyngeal muscle cells. Intestinal expression was strongest in early larval stages (L1-L3). Spermathecal expression was extremely strong in both L4s and adults. We also examined the expression of this reporter construct in several mutant strains and found there were no differences in the *tat-2* expression patterns in *elo-5(gk208)*, *tat-2(tm2332)* or *tat-2(ku449);elo-5(gk208)* worms. In addition, no difference in the expression was found when we varied the temperatures, mmBCFA supplementation, or salt (data not shown).

Based on the expression data, we examined a *tat-2* deletion strain for defects specific to the spermatheca, the amphid neurons and the excretory cell. Males from *tat-2(tm2332)* were competent for mating and there were no obvious defects in spermathecal function (data not shown). In addition, we considered that a *tat-2* deletion strain might have defects in amphid structure. However, DiOc staining indicated that there were no defects in dye-uptake by the amphid neurons in *tat-2(tm2332)* and the architecture of the labeled cells appeared normal (data not shown). The sensitivity of *tat-2(tm2332)* to high and low osmolarity was also examined based on the observed excretory cell expression. After ten minutes on high-salt NGM plates (500 mM NaCl), 100% of either control or *tat-2(tm2332)* worms were paralyzed, indicating that there were no drastic differences in the salt tolerance of *tat-2* worms as compared to wild-type worms.

Ectopic expression of *tat-2* does not cause growth defects

Since a mutation in *tat-2* can relieve a growth arrest, it is possible that TAT-2 protein has a general growth suppression function and overexpression of the *tat-2* gene would be harmful to growth. To test this, we fused the rescuing cDNA downstream of the promoter for the heat shock protein hsp-16 (pPD 49.83) and performed experiments on two independent lines harboring integrated copies of this construct. Upon heat shock of various stages from eggs to adults, the *hsp16::tat-2* lines showed no obvious defects at the gross and microscopic level compared to control worms that contained pPD 49.83 alone (data not shown). To confirm that *tat*-2 is overexpressed in these lines, we performed quantitative RT-PCR with one of the lines to check *tat-2* mRNA levels. The *hsp-16::tat-2* line had approximately 2.5 times the amount of *tat-2* mRNA post-heat shock when compared to the control (data not shown). This indicates that an increase in *tat-2* expression at this level is not sufficient to induce any visible growth suppression.
